# Preparation and characterization of Pd supported on 5-carboxyoxindole functionalized cell@Fe_3_O_4_ nanoparticles as a novel magnetic catalyst for the Heck reaction

**DOI:** 10.1039/d0na00954g

**Published:** 2021-03-11

**Authors:** Majid Mohammadnia, Nazanin Poormirzaei

**Affiliations:** Department of Chemistry, Islamic Azad University Iran Majidmohammadnia.chem@gmail.com; Department of Chemistry, Mashhad Branch, Islamic Azad University Iran

## Abstract

Pd supported on 5-carboxyoxindole functionalized cell@Fe_3_O_4_ nanoparticles (Pd@CAI@cell@Fe_3_O_4_), a new magnetic nanocatalyst, was prepared and characterized using inductively coupled plasma atomic emission spectroscopy, Fourier-transform infrared spectroscopy, X-ray diffraction, scanning electron microscopy, transmission electron microscopy, thermogravimetric analysis, and energy-dispersive X-ray spectroscopy techniques. The synthesized nanocatalyst (Pd@CAI@cell@Fe_3_O_4_) was employed for Heck-type arylation of different substituted maleimides with iodoarenes in good to excellent yields. This green catalyst was easily recovered and reused several times with no substantial loss of activity, providing a clean and efficient synthetic procedure with excellent yield and reduced time.

## Abstract 2

Pd supported on 5-carboxyoxindole functionalized cell@Fe_3_O_4_ nanoparticles (Pd@CAI@cell@Fe_3_O_4_), a new magnetic nanocatalyst, was prepared and characterized.

## Graphical abstract

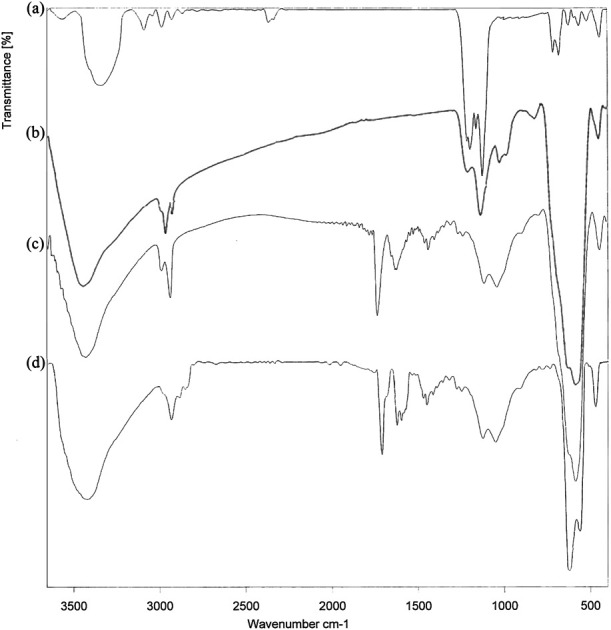

## 1. Introduction

Nanocatalysis has emerged as a field at the interface between homogeneous and heterogeneous catalysis and suggests exceptional solutions to the various industries for catalyst enhancement.^1,2^ In the meantime, heterogeneous catalysis is one of the oldest commercial implementations of nanoscience, and nanoparticles of metals, semiconductors, oxides, and other compounds have been extensively used for significant chemical reactions. The principal focus of this field is the evolution of catalysts that include both metal nanoparticles and a nanomaterials as the main support. In fact, these nanocatalysts have high specific surface area and surface energy, which result in their high catalytic activity. Also, they have other properties, such as improving the selectivity of the reactions while reducing the reaction temperature, minimizing side reactions, and with higher recycling rates. Hence, these catalysis are of great interest for researchers in the synthesis of many organic compounds.^3^

Recently, the majority of scientists increasingly employ biopolymer supports from renewable, durable, and abundant resources as attractive materials for catalysis reactions.^4,5^

Nanocellulose is the most noteworthy and abundant biopolymer, and it is obtained from plants, bacteria and algae; its known properties include hydrophobicity, biodegradability, economy, biocompatibility, and wide chemical-functionalization capacity.^6^

These abilities of cellulose make it an interesting support, and its application as an efficient support for the catalytic processes in the synthesis of many organic compounds has been studied.^6^ In fact, supported nanocellulose is emerging as an attractive protocol to stabilize some transition metal complexes.^6–12^ In recent times, nanocellulose supporting palladium, platinum, zirconium, copper and nickel nanoparticles have been studied and reported.^13–16^

Also, nanocellulose is one of the most perfect coating supports for magnetic nanoparticles (MNPs) due to its ability to not only stabilize nanoparticles in solution but also promote functionalization to produce biopolymer-based catalysts.^17^

In fact, MNPs have attracted great attention due to their unique properties, including eco-friendliness, high flexibility, low toxicity, low Curie temperature, easy preparation and functionalization, large surface-to-volume ratio, facile separation using an external magnet, and a high degree of chemical stability.^18,19^ The design of new, magnetically separable systems has generated a lot of attention in recent years as an attractive candidate to improve the efficient separation of heterogeneous nanocatalysts from products by their response to an external magnetic field.^20,21^ In other words, in order to control the fast oxidation and the tendency of MNPs to agglomerate, their surface is generally protected with organic, inorganic or bio-polymeric materials to form core shell structures, which have thus been named bio-based magnetic nanocatalysts.^22^

Heck cross-coupling reactions^23–25^ involve the coupling of an unsaturated halide with an alkene in the presence of palladium catalyst (or palladium nanomaterial-based catalyst) to form a substituted alkene; they are also referred to as Mizoroki–Heck reactions.^26^ The first work of Heck-type coupling was reported by Hacksell and Daves in 1985,^27^ with a new update in 1990.^28^ In fact, Heck reactions are very significant in industry, since substitution reactions can be accomplished on planar centers.^29^ The first time the Heck reaction was discovered it was by the American chemist Richard F. Heck,^29^ and also Mizoroki, who considerably developed this reaction in organic chemistry.^30^

The C–H functionalization of alkenes is presently under intensive investigation as a direct carbon–carbon bond-forming procedure for the preparation of higher substituted alkenes. The prevalently used Heck reaction enables direct C–H to C–C functionalization of alkenes with aryl and vinyl halides, but is rarely performed with alkyl halide coupling partners due to the propensity of the alkyl group to undergo β-hydride elimination.^31,32^

**Scheme 1:**
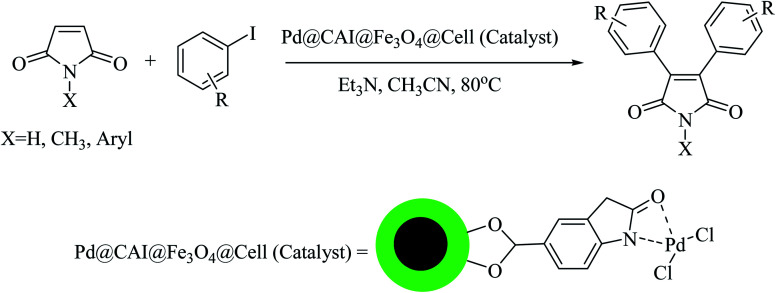
Heck-type arylation with Pd@CAI@cell@Fe_3_O_4_ as a nanomagnetic catalyst.

In continuation of our research on nanomagnetic supports,^33–35^ according to the above-mentioned explanations, in this work, the connected palladium supported on 5-carboxyoxindole functionalized cell@Fe_3_O_4_ (Pd@CAI@cell@Fe_3_O_4_), an efficient and interesting bio-based magnetic nanocatalyst, was prepared. Then, this nanocatalyst was employed for the Heck-type arylation of maleimides with iodoarenes (Scheme 1).

## 2. Experimental

### 2.1. Chemicals and apparatus

Powder X-ray diffraction (PXRD) of the prepared catalyst was performed on a Philips PW 1830 X-ray diffractometer using a Cu Kα source (*λ* = 1.5418 Å) in a Bragg's angle range of 10–80° at 25 °C. Fourier-transform infrared (FT-IR) spectroscopy was carried out using a FT-IR spectrometer (Vector 22, Bruker) in the range of 400–4000 cm^−1^ at room temperature. Scanning electron microscopy (SEM) analysis was recorded using a VEGA//TESCAN KYKY-EM 3200 microscope (acceleration voltage 26 kV). Transmission electron microscopy (TEM) experiments were done on a Philips EM 208 electron microscope. The elemental analysis spectrum of the catalyst was assessed by energy dispersive X-ray (EDX) spectroscopy (VEGA3 XUM/TESCAN). Thermogravimetric analysis (TGA) was performed on a Stanton Red craft STA-780 (London, UK). Nuclear magnetic resonance (NMR) spectra were measured using a Bruker DRX-400 AVANCE instrument (300.1 MHz for ^1^H, 75.4 MHz for ^13^C) in DMSO-d_6_ as a solvent. Magnetic measurements were carried out using a vibration sample magnetometer (VSM, MDK, and Model 7400). The metal loading was determined by inductively coupled plasma-atomic emission spectrometry (ICP-AES). Melting points were evaluated on electrothermal 9100 apparatus.

### 2.2. General procedure

#### 2.2.1. Preparation of cellulose nanocrystals (cell)

To prepare cellulose nanocrystals (cell), first, acidic hydrolysis of Whatman filter paper was employed as reported in the literature with a slight modification. Hydrolysis of the cellulose was obtained after 3 h at 100 °C using 100 mL of 2.5 M HBr and alternating ultrasonication. After dilution with twice-distilled water, the mixture was subjected to five washing/centrifugation cycles to remove excess acid and water-soluble residues. After neutralization to around pH 5, the fine cellulose nanoparticles started to disperse into the aqueous supernatant and were collected by centrifugation at 12 000 rpm for 60 min to remove the ultrafine particles.

#### 2.2.2. Preparation of cell@Fe_3_O_4_

**Scheme 2:**
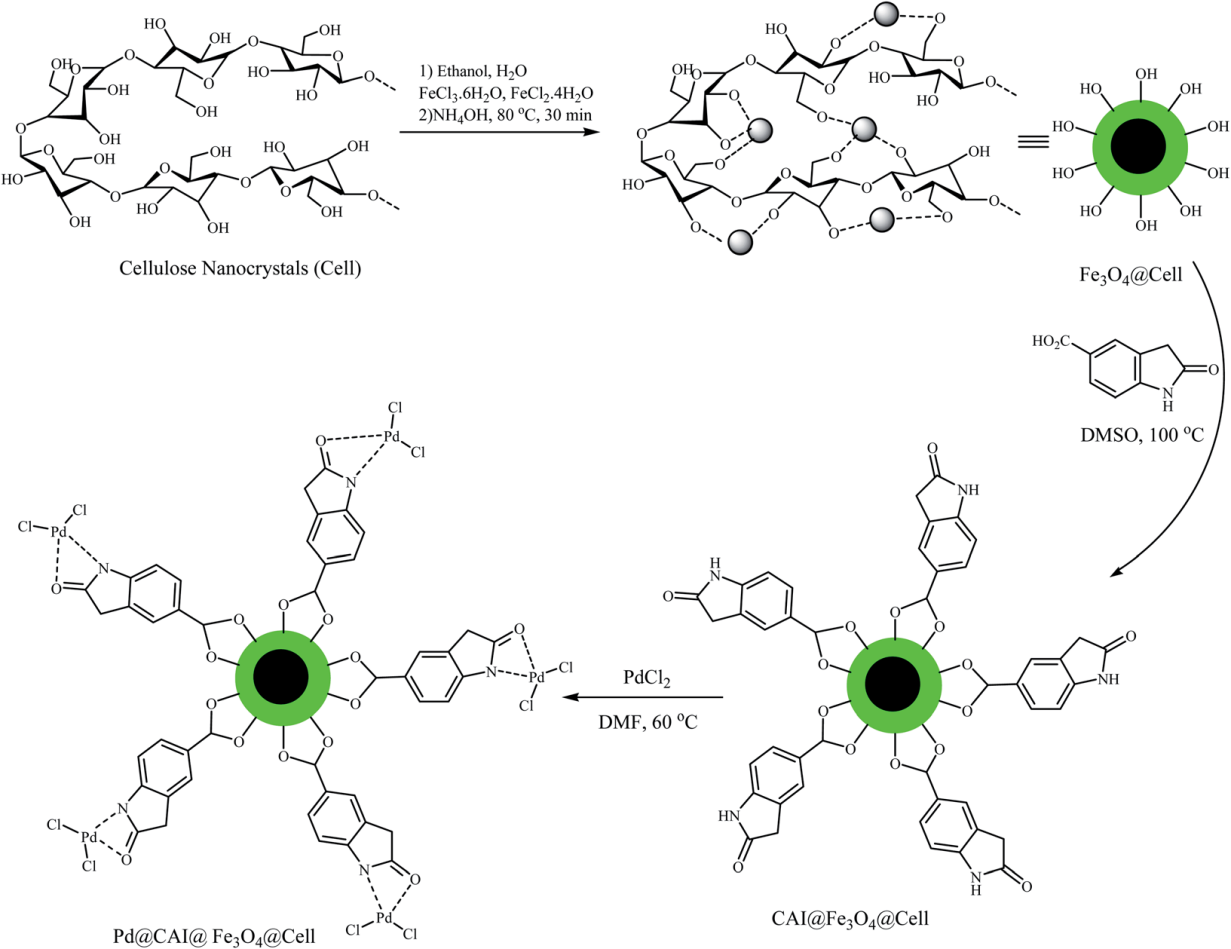
Preparation of the Pd@CAI@cell@Fe_3_O_4_ nanomagnetic catalyst.

Here, nanocellulose (5.0 g) was first added to a solution containing FeCl_3_·6H_2_O (4.865 g, 0.018 mol), FeCl_2_·4H_2_O (1.789 g, 0.0089 mol) and 100 cm^3^ of CH_3_OH/deionized water (50/50), then stirred for 3 h. Next, 10 mL of 25% NH_4_OH (10 mL) was added immediately into the reaction mixture in one portion under a N_2_ atmosphere at 80 °C, followed by vigorous stirring for around 30 min using a magnetic stirrer. The product was washed with deionized water four times (Scheme 2).

#### 2.2.3. Preparation of CAI@cell@Fe_3_O_4_

400 mg of Fe_3_O_4_@cell was mixed with 400 mg of 5-carboxyoxindole (CAI) in 20 mL of DMSO and stirred for 24 h at 100 °C. After the reaction was completed, the mixture was cooled to room temperature, and the obtained precipitate was filtered using an external magnet and rinsed with ethanol several times, then placed in an oven for 24 h to dry, thus the CAI@ Fe_3_O_4_@cell nanoparticles were obtained (Scheme 2).

#### 2.2.4. Preparation of Pd@CAI@cell@Fe_3_O_4_ nanomagnetic catalyst

To prepare the catalyst, 0.50 g of CAI@Fe_3_O_4_@cell in DMF was added to a solution of Pd(Cl)_2_ (0.10 g, 0.45 mmol) in 10 mL DMF under N_2_ atmosphere, and the obtained mixture was stirred for 24 h at 60 °C. After the reaction was completed, the mixture was cooled to room temperature, and the resulting product was collected using an external magnet. The solid black product was washed carefully with deionized water (3 × 25 mL), absolute ether (2 × 25 mL) and absolute ethyl alcohol (2 × 25 mL), then dried in a vacuum oven at room temperature (Scheme 2).

### 2.3. Pd@CAI@cell@Fe_3_O_4_-catalyzed Heck-type arylation of maleimides with iodoarenes

A vial equipped with a stirrer bar was charged with different substituted maleimides (0.1 mmol, 1.0 eq.), iodoarene (0.2 mmol, 2.0 eq.), triethylamine (2.0 eq.) and Pd@CAI@cell@Fe_3_O_4_ (10 mol%). Acetonitrile (10 mL) was then added, and the reaction mixture was vigorously stirred at 80 °C for different lengths of time, according to each substrate. Upon completion of the reaction (as monitored by thin layer chromatography (TLC)), the reaction mixture was cooled to room temperature. After that, the reaction mixture was dissolved in dichloromethane (10 mL) and, subsequently, the Pd@CAI@cell@Fe_3_O_4_ nanoparticle catalyst was separated by an external magnet at 5 min. Removal of the solvent under reduced pressure yielded a crude mixture, which was purified by column chromatography (hexanes/EtOAc gradient) to provide the desired product (Scheme 1).

Spectroscopic data for the unknown products are as follows:

1-Methyl-3,4-diphenyl-1*H*-pyrrole-2,5-dione (Table 2, entry 3): ^1^H NMR (400.13 MHz, CDCl_3_): *δ* = 3.30 (3H, s, N–CH_3_), 7.29–7.33 (m, 4H, Ar), 7.47–7.52 (m, 6H, Ar) ppm. ^13^C NMR (100.6 MHz, CDCl_3_): 24.1, 128.2, 128.3, 128.9, 136.6, 170.2 ppm.

3,4-Bis(4-methoxyphenyl)-1-methyl-1*H*-pyrrole-2,5-dione (Table 2, entry 5): ^1^H NMR (400.13 MHz, CDCl_3_): *δ* = 3.31 (3H, s, N–CH_3_), 3.81 (s, 6H, 2OCH_3_), 6.89–6.95 (m, 4H, Ar), 7.27–7.31 (m, 4H, Ar) ppm. ^13^C NMR (100.6 MHz, CDCl_3_): 24.2, 54.7, 112.2, 119.9, 131.2, 133.4, 159.2, 170.2 ppm.

## 3. Results and discussion

### 3.1. Characterization of the prepared Pd@CAI@cell@Fe_3_O_4_ nanoparticles

#### 3.1.1. FT-IR spectroscopic analysis

**Fig. 1:**
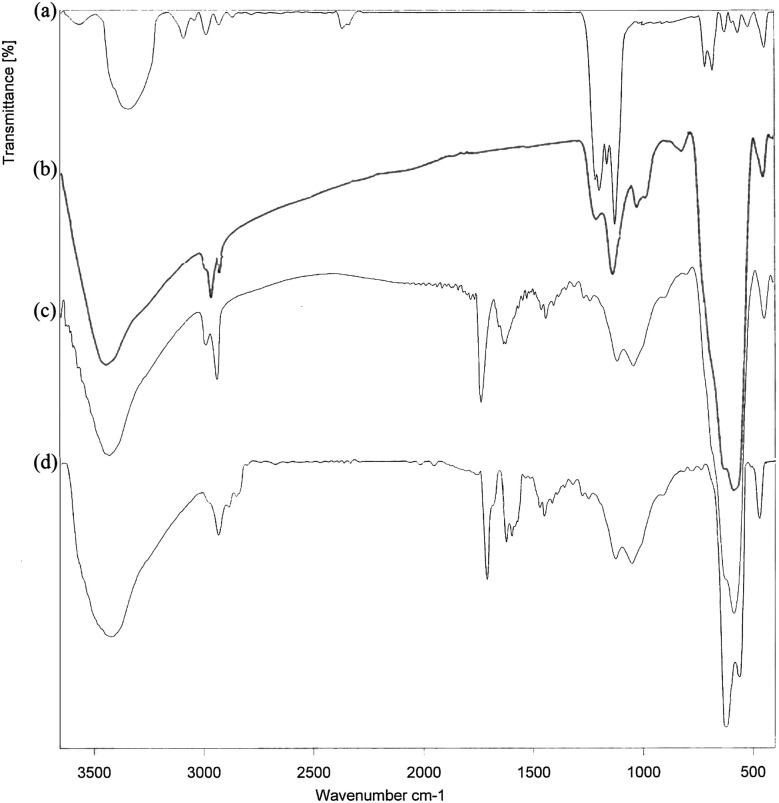
The FT-IR spectra of the (a) cell, (b) cell@Fe_3_O_4_, (c) CAI@cell@Fe_3_O_4_ and (d) Pd@CAI@cell@Fe_3_O_4_.

The FT-IR spectra of cell, cell@Fe_3_O_4_, CAI@cell@Fe_3_O_4_ and Pd@CAI@cell@Fe_3_O_4_ are displayed in Fig. 1. As can be seen in Fig. 1 for the nanocell, the adsorption peak at 1058 cm^−1^ exhibits vibration of the C–O–C in the pyranone ring. The peaks for C–H and C–O vibrations in the polysaccharide rings of cellulose are around 1200–1050 cm^−1^. The absorption bands at 3420 cm^−1^ and 2998–2850 cm^−1^ are attributed to the O–H and C–H stretching vibrations, respectively. Also, the adsorption bands at 586 and 520 cm^−1^ are ascribed to Fe–O stretching band, and those at 3442 cm^−1^ correspond to broad OH groups on the magnetic surface of the MNPs.

After synthesis of CAI@cell@Fe_3_O_4_, two absorption peaks were observed at 2990 and 2930 cm^−1^, which can be assigned to symmetric stretching of the C–H group. The bands situated at 1460 and 1629 cm^−1^ display symmetric and asymmetric stretching adsorption peaks, and one at 1700 cm^−1^ can be assigned to the C=O group related to 5-carboxyoxindole connected to the cell@Fe_3_O_4_ nanoparticles.

Also, the intensity of the adsorption peak at 586 cm^−1^, which is assigned to the bending vibration of C–H in the pyridine heterocyclic ring, reduces after the formation of CAI@cell@Fe_3_O_4_ complex with Pd. Comparison of FTIR spectra results confirm the well grafted 5-carboxyoxindole (CAI) as ligand connected to the Pd metal on CAI@cell@Fe_3_O_4_.

#### 3.1.2. XRD analysis

**Fig. 2:**
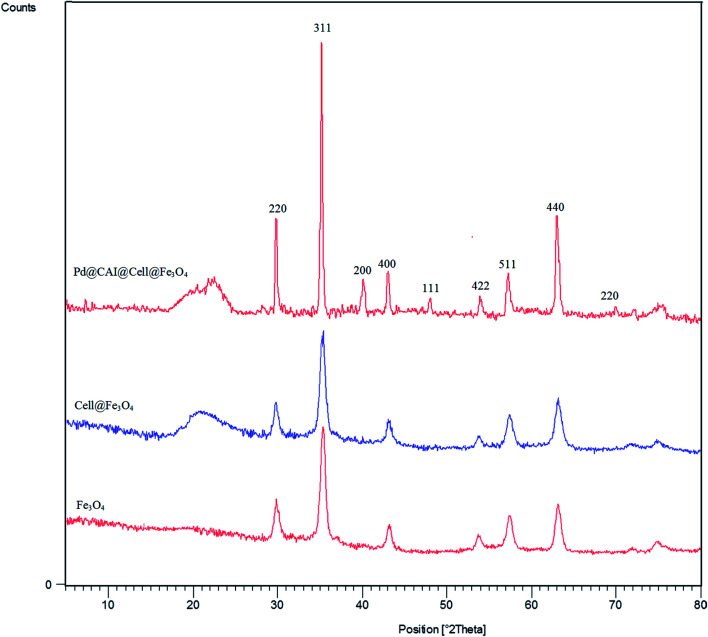
PXRD patterns of Fe_3_O_4_, cell@Fe_3_O_4_ and the Pd@CAI@cell@Fe_3_O_4_ nanoparticles.

The PXRD spectrum of Pd@CAI@cell@Fe_3_O_4_ is shown in Fig. 2. Wide-angle PXRD measurements were performed to affirm the presence of palladium on the CAI@cell@Fe_3_O_4_. The wide peak at 2*θ* = 22.5° present in both the Pd@CAI@cell@Fe_3_O_4_ and cell@Fe_3_O_4_ spectra is concluded to be from nanocellulose. Also, the PXRD pattern revealed that standard Fe_3_O_4_ crystal has six diffraction peaks, namely, (220), (311), (400), (422), (511) and (440) at 2*θ* = 30, 35.5, 43.5, 54, 57, 63 (Fig. 10). Also, the index peaks at 2*θ* = 40.0°, 47° and 68° are assigned to diffractions from various lattice planes, including the (111), (200) and (220) present in cubic palladium. The average Pd@CAI@cell@Fe_3_O_4_ NP diameter was estimated to be approximately 15 nm from the PXRD results by the Scherrer equation, *D* = *kλ*/*β* cos *θ*, where *k* is a constant (generally considered as 0.94), *λ* is the wavelength of Cu Kα (1.54 Å), *β* is the corrected diffraction line full-width at half-maximum (FWHM), and *θ* is Bragg's angle.

#### 3.1.3. Thermogravimetric analysis (TGA)

**Fig. 3:**
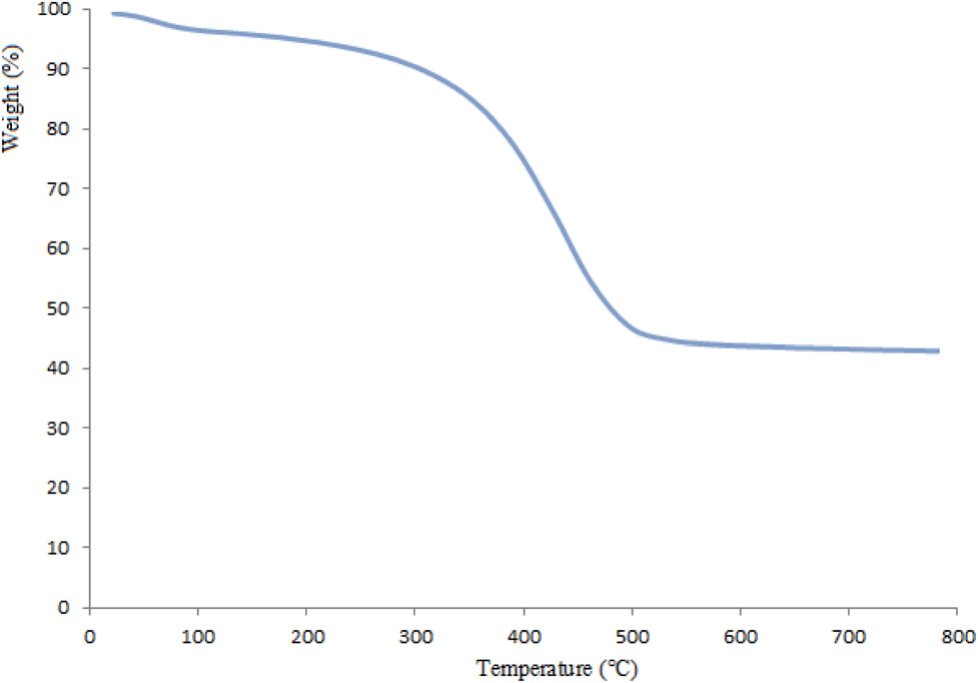
Thermogravimetric analysis of Pd@CAI@cell@Fe_3_O_4_.

TGA was used to determine the thermostability of Pd@CAI@cell@Fe_3_O_4_. As shown in Fig. 3, the first weight loss at almost 100 °C (3%) was allotted to the vaporization of adsorbed water molecules. According to the obtained results, Pd@CAI@cell@Fe_3_O_4_ is stable before 200 °C. The mass weight loss of nearly 50% between 200–500 °C is attributed to thermal decomposition of the organic group.

#### 3.1.4. Scanning electron microscopy (SEM)

**Fig. 4:**
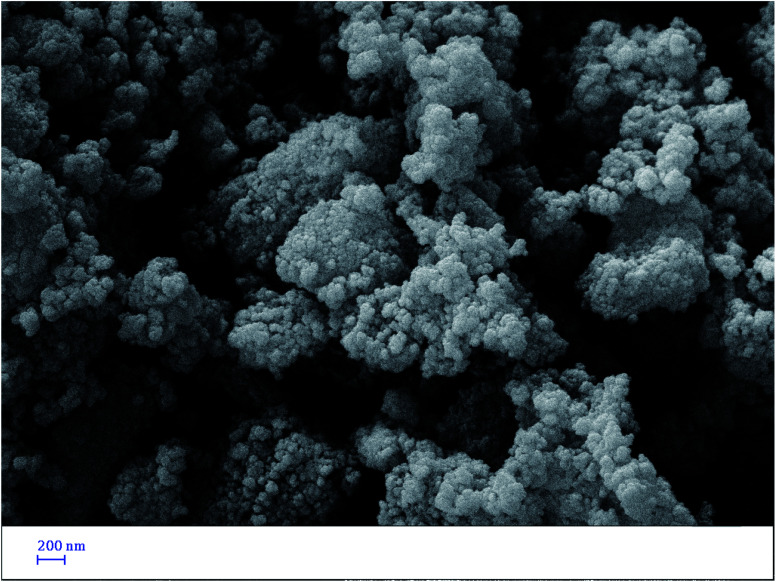
SEM image of Pd@CAI@cell@Fe_3_O_4_.

The SEM image of Pd@CAI@cell@Fe_3_O_4_ is presented in Fig. 4. According to the SEM image, the nanoparticles have a spherical morphology. Besides this, the size distribution is narrow, and the mean size of the nanocomposite is around 15–30 nm.

#### 3.1.5. Transmission electron microscopy (TEM)

**Fig. 5:**
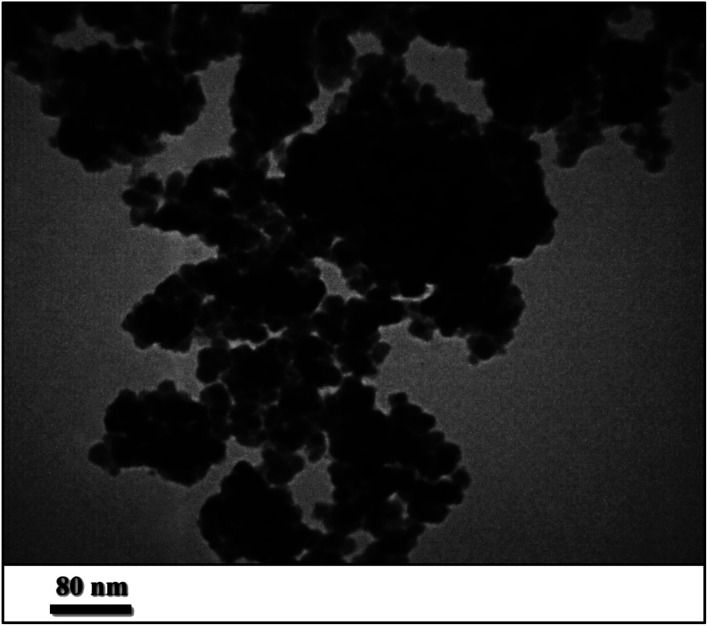
TEM image of Pd@CAI@cell@Fe_3_O_4_.

The morphology of the prepared catalysts consisting of Pd@CAI@cell@Fe_3_O_4_ nanoparticles was studied by TEM, which is displayed in Fig. 5, showing particles have spherical morphology. According to TEM image, the average particle size is estimated to be about 17 nm for Pd@CAI@cell@Fe_3_O_4_ nanoparticles, which is in good agreement with the crystallite size estimated from PXRD, at 15 nm. As shown in Fig. 5, a fundamentally core–shell structure (dark core for Pd and Fe_3_O_4_ nanoparticles, and light shell for the organic group) was concluded. This is a presentation of the almost single crystalline character of the Pd@CAI@cell@Fe_3_O_4_ nanoparticles.

#### 3.1.6. EDX spectroscopy

**Fig. 6:**
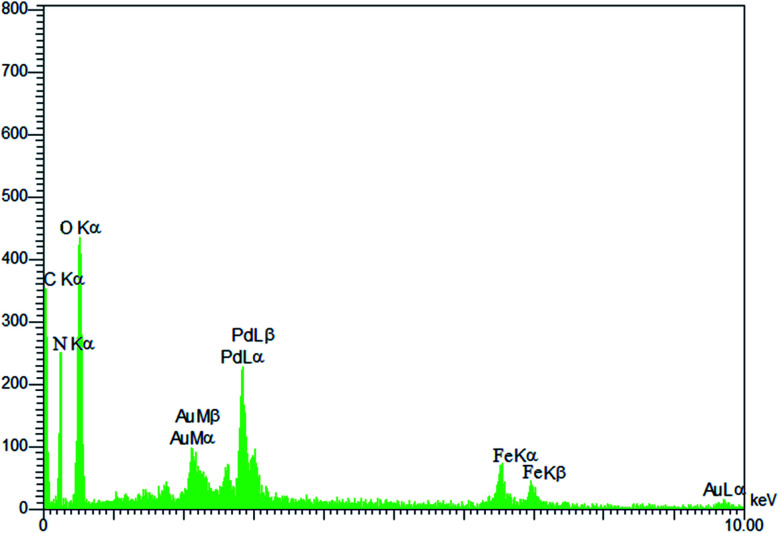
EDX spectrum of Pd@CAI@cell@Fe_3_O_4_.

The EDX analysis of Pd@CAI@cell@Fe_3_O_4_ is shown in Fig. 6. As can be seen, Pd@CAI@cell@Fe_3_O_4_ is composed of Fe, C, O and Pd, indicating that Pd has been inserted in the desired catalyst. In other words, there is a Pd peak, which is consistent with the ICP-AES results.

#### 3.1.7. ICP-AES analysis

The ICP-AES analysis shows the weight percentage of Pd to be 10% in the Pd@CAI@cell@Fe_3_O_4_ NPs.

#### 3.1.8. Magnetic properties of the catalyst

**Fig. 7:**
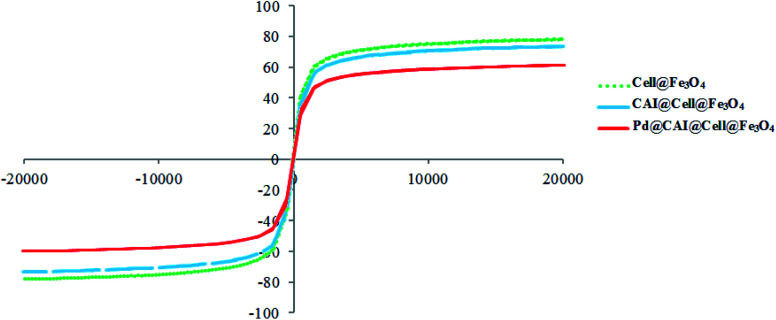
Room-temperature magnetization curves of cell@Fe_3_O_4_, CAI@cell@Fe_3_O_4_ and Pd@CAI@cell@Fe_3_O_4_ nanoparticles.

A VSM was used to characterize the magnetic properties of cell@Fe_3_O_4_, CAI@cell@Fe_3_O_4_ and Pd@CAI@cell@Fe_3_O_4_ at 300 K (Fig. 7). The magnetization curves for these nanoparticles display no hysteresis in their magnetization. As can be seen in Fig. 7, the saturation magnetization values for cell@Fe_3_O_4_, CAI@cell@Fe_3_O_4_ and Pd@CAI@cell@Fe_3_O_4_ are 79.0, 74.1 and 61.1 emu g^−1^, respectively. A small decrease in the saturation magnetization of Pd@CAI@cell@Fe_3_O_4_ originates from the grafting of Pd on the surface of the CAI@cell@Fe_3_O_4_ nanoparticles. In other words, these differences arise from the different coating layers and their thicknesses on the surface of the MNPs. The prepared catalyst reveals excellent magnetic characteristics and thus can be quickly and completely separated from the reaction media using an external magnet.

### 3.2. Catalytic application of Pd@CAI@cell@Fe_3_O_4_ as a nanomagnetic catalyst for the Heck-type arylation of maleimides with iodoarenes

First, to find the optimum conditions, the reaction of maleimides (1 mmol) and 4-iodotoluene (2 mmol) in the presence of the Pd@CAI@cell@Fe_3_O_4_ as nanocatalyst was selected as a model reaction. The reaction was performed using various amounts of Pd@CAI@cell@Fe_3_O_4_ as a catalyst (5, 10 and 15 mol%) at different temperatures (50 and 80 °C) with Et_3_N as a base in various solvents. According to the obtained results, which are reported in Table 1, the optimum conditions were 10 mol% of Pd@CAI@cell@Fe_3_O_4_ as a catalyst and Et_3_N (2.0 eq.) as a base at 80 °C in CH_3_CN (Table 1, entry 10).

**Table 1:** Optimization of the conditions for the Heck-type arylation of maleimide with 4-iodotoluene in the presence of Pd@CAI@cell@Fe_3_O_4_ as a nanocatalyst

Entry	Solvent	Temperature (°C)	Catalyst (mol%)	Base	Time (h)	Yield^a^ (%)
1	DMF	80	15	Et_3_N	12	28
3	H_2_O	80	15	Et_3_N	12	26
4	Ethanol	80	15	Et_3_N	12	32
5	CH_3_CN	80	15	Et_3_N	12	85
6	CH_3_CN	50	5	Et_3_N	15	38
7	CH_3_CN	50	10	Et_3_N	15	41
8	CH_3_CN	50	15	Et_3_N	15	45
9	CH_3_CN	80	5	Et_3_N	12	63
10	CH_3_CN	80	10	Et_3_N	12	84
11	CH_3_CN	80	15	Et_3_N	12	85
12	Solvent free	80	15	Et_3_N	12	42
13	CH_3_CN	80	Fe_3_O_4_ (15)	Et_3_N	12	Trace
14	CH_3_CN	80	Cell@Fe_3_O_4_ (15)	Et_3_N	12	Trace
15	CH_3_CN	80	CAI@cell@Fe_3_O_4_ (15)	Et_3_N	12	Trace
16	CH_3_CN	80	Catalyst free	Et_3_N	12	—

a Yields refer to isolated pure product.

Afterwards, different substituted maleimides and iodoarene derivatives were applied in the Heck-type arylation to prepare the corresponding products, which led to high to excellent yields (Table 2). As can be observed from Table 2, the starting materials, either with electron-donating or electron-withdrawing substituents on the maleimides and iodoarenes, provided the desirable products in high to excellent yields (Table 2).

**Table 2:** One-pot reactions of the Heck-type arylation of substituted maleimides with iodoarenes in the presence of Pd@CAI@cell@Fe_3_O_4_ (10 mol%) as a nanocatalyst at 80 °C in CH_3_CN (10 mL)

Entry	I–Ar–R	Substituted maleimides	Time (h)	Yield (%)	Melting point (°C)	
					Found	Reported [ref.]
1	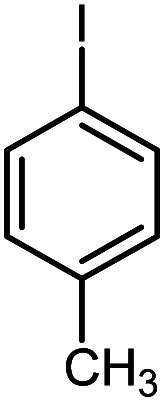	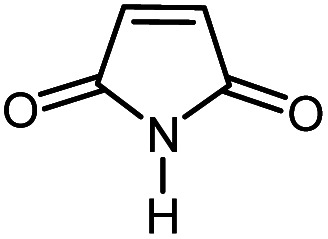	12	84	175–176	172–174 [ref. 36]
2	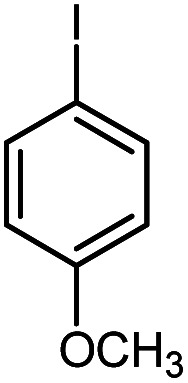	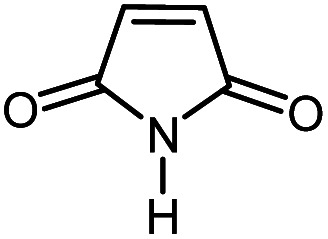	12	87	200–201	200–201 [ref. 36]
3	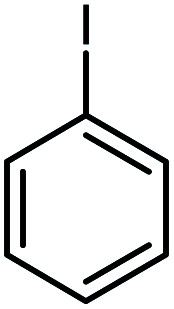	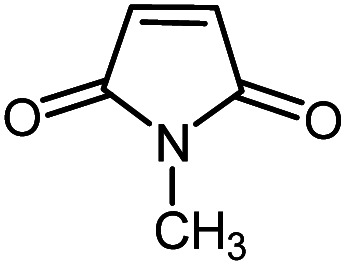	14	84	121–122	118–120 [ref. 36]
4	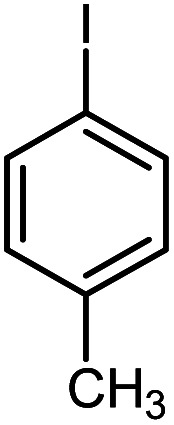	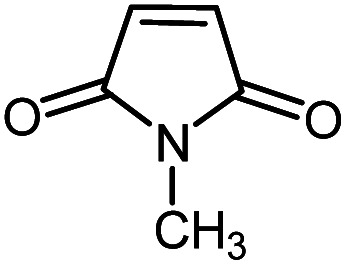	12	85	178–179	178–180 [ref. 36]
5	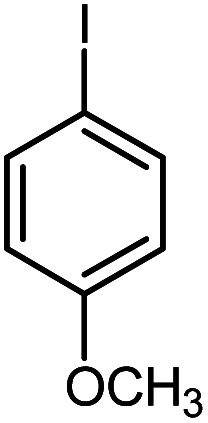	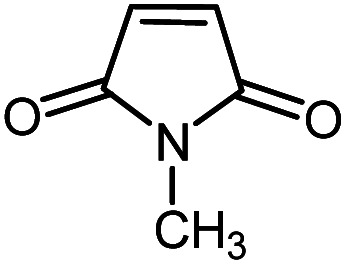	11	85	131	130–132 [ref. 36]
6	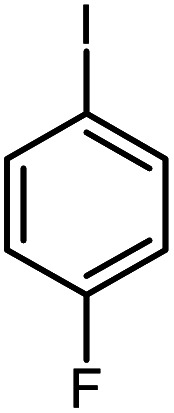	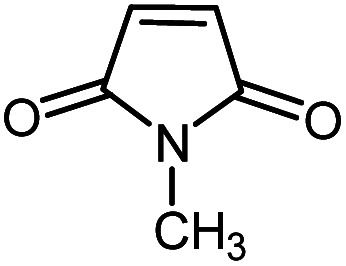	14	77	176–178	176–178 [ref. 36]
7	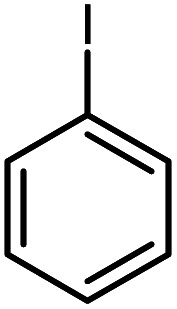	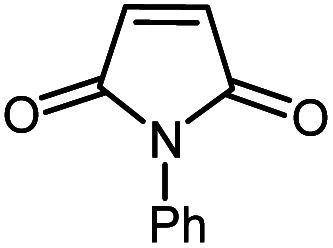	13	84	152–153	152–154 [ref. 36]
8	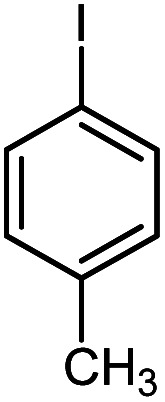	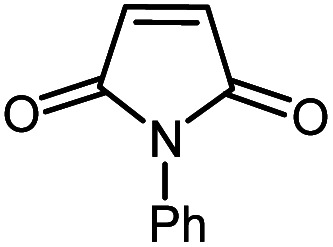	12	85	159–160	158–159 [ref. 36]
9	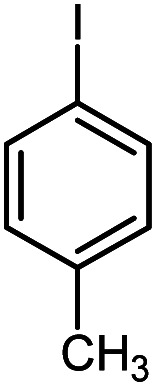	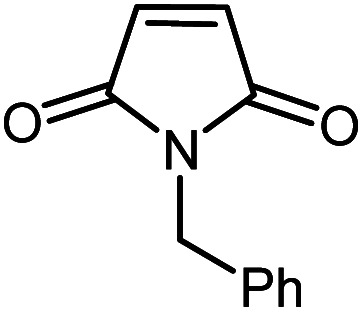	13	84	130–131	131–132 [ref. 36]
10	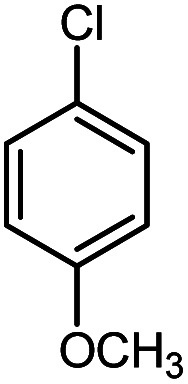	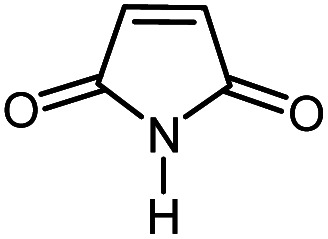	11	86	202	200–201 [ref. 36]
11	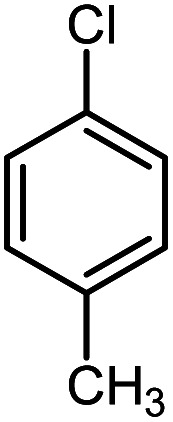	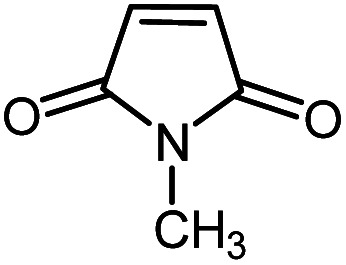	12	87	180	178–180 [ref. 36]
12	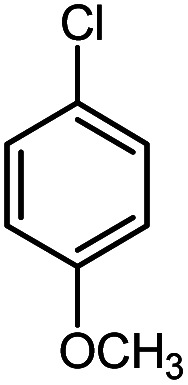	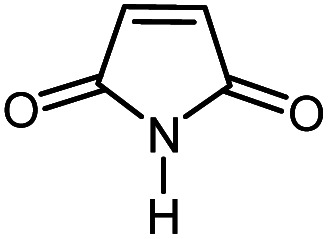	12	88	198–199	200–201 [ref. 36]
13	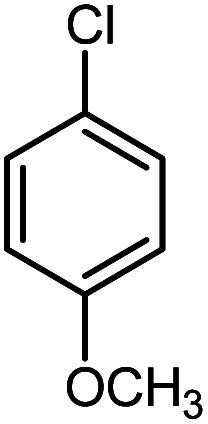	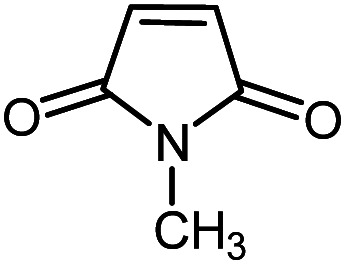	12	84	133	130–132 [ref. 36]
14	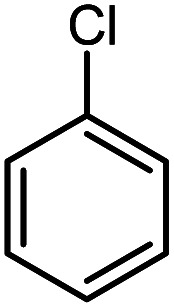	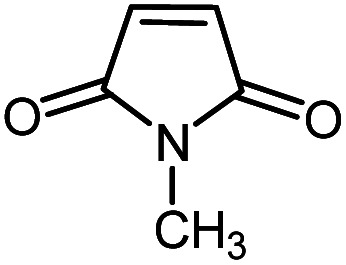	13	85	119–121	118–120 [ref. 36]

In order to show the accessibility of the present work in comparison with the only reported result in the literature, we summarized some of the results for the Heck-type arylation of maleimide with 4-iodotoluene. The results show that the Pd@CAI@cell@Fe_3_O_4_ (10 mol%) at 80 °C (reaction time 12 h and yield 84%) is the better catalyst relative to PdCl_2_ (10% mol) at 100 °C (reaction time 24 h and yield 28%)^36^ due to the reusability of the catalyst several times with no substantial loss of activity, its short time reaction and good yields of the product.

**Fig. 8:**
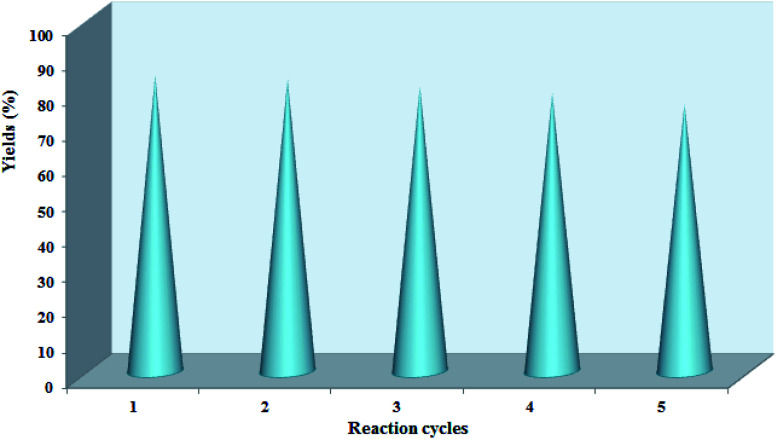
Recycling of the Pd@CAI@cell@Fe_3_O_4_ as a magnetic nanocatalyst.

Additionally, the recycling and reusability of the Pd@CAI@cell@Fe_3_O_4_ nanoparticles were studied using a model reaction (Experimental section). According to the observed results, the recovered catalyst was reused for five runs with no loss of activity (Fig. 8).

**Fig. 9:**
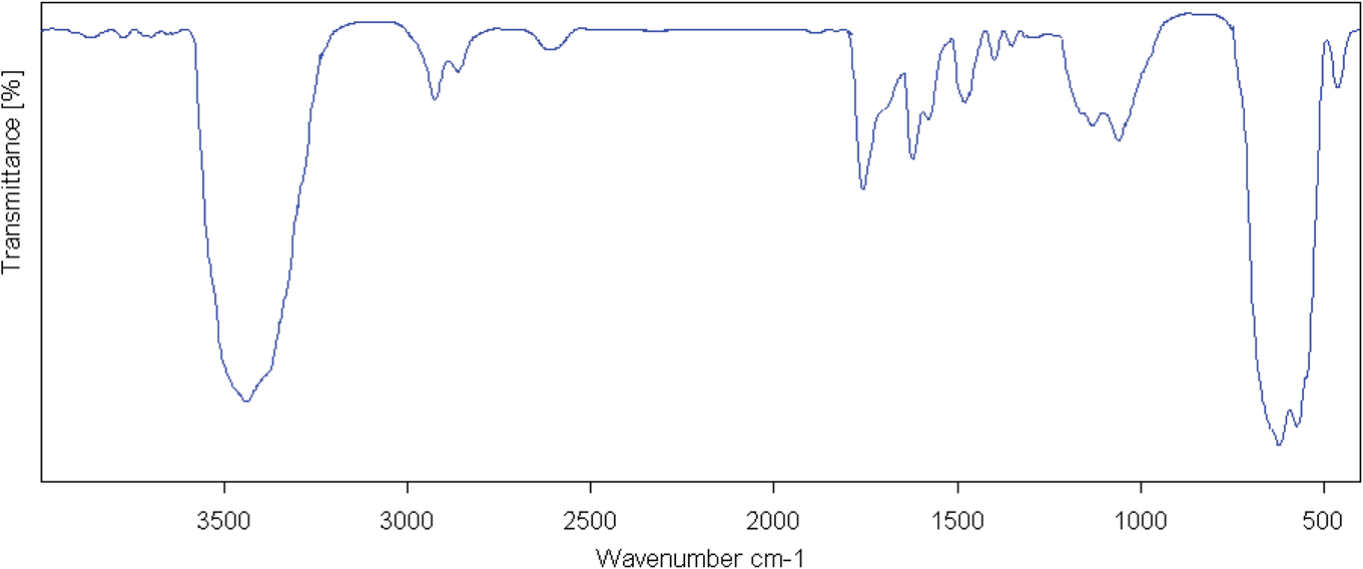
FT-IR analysis of the Pd@CAI@cell@Fe_3_O_4_ nanoparticles after recycling them five times.

**Fig. 10:**
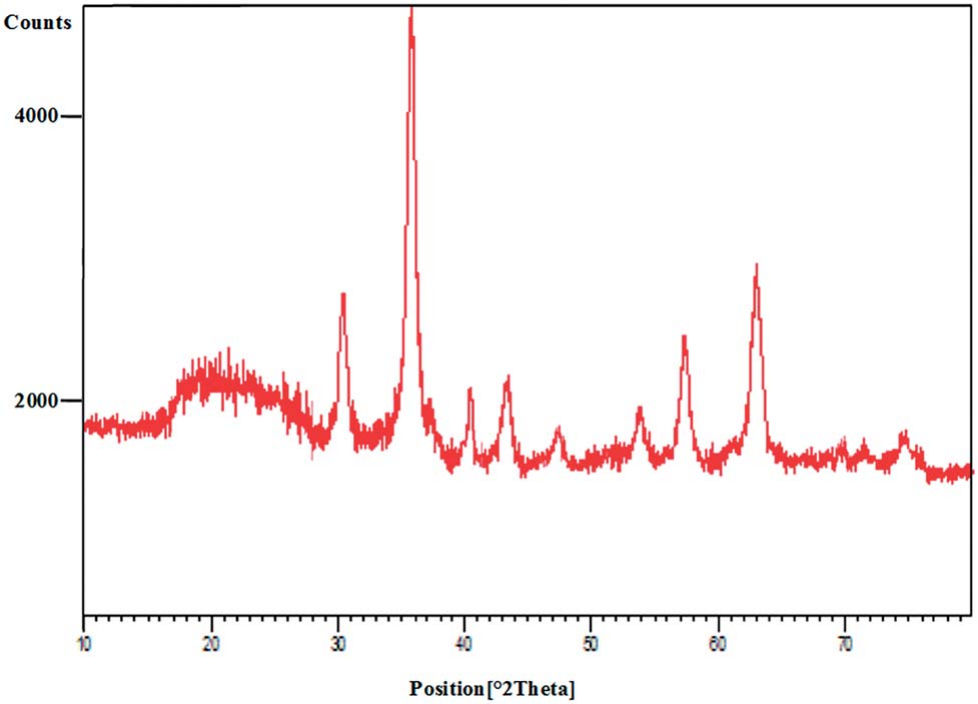
PXRD analysis of Pd@CAI@cell@Fe_3_O_4_ nanoparticles after recycling them five times.

Moreover, the FT-IR, PXRD and ICP analysis of recycled Pd@CAI@cell@Fe_3_O_4_ (after recycling five times) were conducted, as shown in Fig. 9 and 10. The ICP-AES analysis shows the weight percentage of the Pd to be 10% in the Pd@CAI@cell@Fe_3_O_4_ NPs. As can be seen, the structure of the recycled nanocatalyst has not changed and is quite similar to that of the newly prepared catalyst.

## 4. Conclusions

In conclusion, we report the preparation of Pd supported on 5-carboxyoxindole functionalized cell@Fe_3_O_4_ nanoparticles (Pd@CAI@cell@Fe_3_O_4_) as an efficient, novel and reusable heterogeneous nanomagnetic catalyst. The prepared nanomagnetic catalyst was characterized by XRD, ICP-AES, SEM, TEM, FT-IR, TGA, VSM and EDX techniques and was used successfully for the Heck-type arylation between different substituted maleimides with iodoarenes. The Pd@CAI@cell@Fe_3_O_4_ nanomagnetic catalyst demonstrated an average particle size of about 15 nm. The nanocatalyst was recovered by simple separation using an external magnet and reused for subsequent cycles. The prepared nanocatalyst exhibited several advantages, including high specific surface area, more active sites, prominent chemical and thermal stability, decrease in the leaching (the release) of the nanocatalyst into the bioenvironment (ecosystem), the presence of organic groups for easier modification, and lower accumulation with respect to other nanocatalysts.

## Conflicts of interest

There are no conflicts to declare.

## Supplementary Material

## References

[cit1] Cong H., Porco Jr. J. A. (2012). Chemical synthesis of complex molecules using nanoparticle catalysis. ACS Catal..

[cit2] Tao L., Bi X., Zhang L., Chen G., Zhao P., Yang J. L., Meng X. (2020). Na-doped OMS-2-catalyzed highly selective aerobic oxidation of ethyl lactate to ethyl pyruvate under mild conditions. Appl. Catal., A.

[cit3] Sharma R. K., Dutta S., Sharma S., Zboril R., Varma R. S., Gawande M. B. (2016). Fe_3_O_4_ (iron oxide)-supported nanocatalysts: synthesis, characterization and applications in coupling reactions. Green Chem..

[cit4] Meng X., Bi X., Yu C., Chen G., Chen B., Jing Z., Zhao P. (2018). Ball-milling synthesized hydrotalcite supported Cu–Mn mixed oxide under solvent-free conditions: an active catalyst for aerobic oxidative synthesis of 2-acylbenzothiazoles and quinoxalines. Green Chem..

[cit5] Meng X., Zhang J., Chen B., Jing Z., Zhao P. (2016). Copper supported on H^+^-modified manganese oxide octahedral molecular sieves (Cu/H-OMS-2) as a heterogeneous biomimetic catalyst for the synthesis of imidazo[1,2-*a*]-N-heterocycles. Catal. Sci. Technol..

[cit6] Cirtiu C. M., Dunlop-Briere A. F., Moores A. (2011). Cellulose nanocrystallites as an efficient support for nanoparticles of palladium: application for catalytic hydrogenation and Heck coupling under mild conditions. Green Chem..

[cit7] Xu Y., Zhang L., Cui Y. (2008). Catalytic performance of cellulose supported palladium complex for Heck reaction in water. J. Appl. Polym. Sci..

[cit8] Reddy K. R., Kumar N. S., Reddy P. S., Sreedhar B., Kantam M. L. (2006). Cellulose supported palladium(0) catalyst for Heck and Sonogashira coupling reactions. J. Mol. Catal. A: Chem..

[cit9] Habibi Y., Lucia L. A., Rojas O. J. (2010). Cellulose nanocrystals: chemistry, self-assembly, and applications. Chem. Rev..

[cit10] Siqueira G., Bras J., Dufresne A. (2010). Cellulosic bionanocomposites: a review of preparation, properties and applications. Polymers.

[cit11] Zhang J., Elder T. J., Pu Y., Ragauskas A. J. (2007). Facile synthesis of spherical cellulose nanoparticles. Carbohydr. Polym..

[cit12] Reddy K. R., Kumar N. S., Sreedhar B., Kantam M. L. (2006). *N*-Arylation of nitrogen heterocycles with aryl halides and arylboronic acids catalyzed by cellulose supported copper(0). J. Mol. Catal. A: Chem..

[cit13] Alesi S., Di Maria F., Melucci M., Macquarrie D. J., Luque R., Barbarella G. (2008). Microwave-assisted synthesis of oligothiophene semiconductors in aqueous media using silica and chitosan supported Pd catalysts. Green Chem..

[cit14] Lipshutz B. H., Butler T., Swift E. (2008). C–C bond formation catalyzed heterogeneously by nickel-on-graphite (Ni/Cg). Org. Lett..

[cit15] Margelefsky E. L., Zeidan R. K., Davis M. E. (2008). Cooperative catalysis by silica-supported organic functional groups. Chem. Soc. Rev..

[cit16] Wang Z., Chen G., Ding K. (2008). Self-supported catalysts. Chem. Rev..

[cit17] Hosseinikhah S. S., Mirjalili B. B. F. (2020). Fe_3_O_4_@NCs/Sb (V): as a cellulose based nano-catalyst for the synthesis of 4*H*-pyrimido[2,1-*b*]benzothiazoles. Polycyclic Aromat. Compd..

[cit18] Salehi N., Mirjalili B. F. (2017). Synthesis of highly substituted dihydro-2-oxopyrroles using Fe_3_O_4_@nano-cellulose–OPO_3_H as a novel bio-based magnetic nanocatalyst. RSC Adv..

[cit19] Wu W., He Q., Jiang C. (2008). Magnetic iron oxide nanoparticles: synthesis and surface functionalization strategies. Nanoscale Res. Lett..

[cit20] Heydari F., Mobinikhaledi A., Zolfigol M. A. (2020). Synthesis of a novel Pd supported polymeric magnetic nanoparticles with urea–pyridine bridge: application as an efficient catalyst for the C–C and C–N bond formation. J. Porous Mater..

[cit21] Mohammadi L., Zolfigol M. A., Khazaei A., Yarie M., Ansari S., Azizian S., Khosravi M. (2018). Synthesis of nanomagnetic supported thiourea–copper(i) catalyst and its application in the synthesis of triazoles and benzamides. Appl. Organomet. Chem..

[cit22] Azad S., Mirjalili B. F. (2016). Fe_3_O_4_@nano-cellulose/TiCl: a bio-based and magnetically recoverable nano-catalyst for the synthesis of pyrimido[2,1-*b*]benzothiazole derivatives. RSC Adv..

[cit23] Heck R. F. (1968). Acylation, methylation, and carboxyalkylation of olefins by group VIII metal derivatives. J. Am. Chem. Soc..

[cit24] Dieck H. A., Heck R. F. (1974). Organophosphinepalladium complexes as catalysts for vinylic hydrogen substitution reactions. J. Am. Chem. Soc..

[cit25] Heck R. F. (1982). Palladium-catalyzed vinylation of organic halides. Org. React..

[cit26] Mizoroki T., Mori K., Ozaki A. (1971). Arylation of olefin with aryl Iodide catalyzed by palladium. Bull. Chem. Soc. Jpn..

[cit27] Hacksell U., Daves Jr G. D. (1985). The chemistry and biochemistry of C-nucleosides and C-aryglycosides. Prog. Med. Chem..

[cit28] Daves Jr G. D. (1990). C-glycoside synthesis by palladium-mediated glycal–aglycon coupling reactions. Acc. Chem. Res..

[cit29] Heck R. F., Nolley J. P. (1972). Palladium-catalyzed vinylic hydrogen substitution reactions with aryl, benzyl, and styryl halides. J. Org. Chem..

[cit30] Mizoroki T., Mori K., Ozaki A. (1972). Dimerization of ethylene catalyzed by σ-aryl nickel compound in the presence of trifluoroboron etherate. Bull. Chem. Soc. Jpn..

[cit31] Liwosz T. W., Chemler S. R. (2012). Copper-catalyzed enantioselective intramolecular alkene amination/intermolecular Heck-type coupling cascade. J. Am. Chem. Soc..

[cit32] Wellington K. W., Benner S. A. (2006). A review: synthesis of aryl C-glycosides via the Heck coupling reaction. Nucleosides, Nucleotides Nucleic Acids.

[cit33] Asghari S., Mohammadnia M. (2017). Synthesis and characterization of pyridine-4-carboxylic acid-functionalized Fe_3_O_4_ nanoparticles as a magnetic catalyst for the synthesis of tetrahydrobenzo[*b*]pyran derivatives under solvent-free conditions. Inorg. Nano-Met. Chem..

[cit34] Asghari S., Mohammadnia M. (2016). Synthesis and characterization of pyridine-4-carboxylic acid functionalized Fe_3_O_4_ nanoparticles as a magnetic catalyst for synthesis of pyrano[3,2-*b*]pyranone derivatives under solvent-free conditions. Res. Chem. Intermed..

[cit35] Shaterian H. R., Mohammadnia M. (2015). Mild preparation of 2-amino-3-cyano-4-aryl-4*H*-benzo[*h*]chromenes and 2-amino-3-cyano-1-aryl-1*H*-benzo[*f*]chromenes, under solvent-free conditions, catalyzed by recyclable basic ionic liquids. Res. Chem. Intermed..

[cit36] Jafarpour F., Shamsianpour M., Issazadeh S., Dorrani M., Hazrati H. (2017). Palladium-catalyzed direct arylation of maleimides: a simple route to bisaryl-substituted maleimides. Tetrahedron.

